# Assessing museums’ daylighting adequacy without annual measurement campaign: Dataset of a confrontation between measured and simulated illuminance values inside the Cetacean Gallery of the Charterhouse of Calci

**DOI:** 10.1016/j.dib.2020.106065

**Published:** 2020-07-24

**Authors:** Francesco Leccese, Giacomo Salvadori, Giuseppe Tambellini, Zehra Tugce Kazanasmaz

**Affiliations:** a School of Engineering, University of Pisa, Italy; b Dept. of Architecture, Izmir Institute of Technology, Turkey

**Keywords:** Daylighting simulations accuracy, Annual measurement campaign, Climate-based daylighting model, Cultural heritage lighting

## Abstract

Lighting Cultural Heritage is a complex task: light is necessary for the act of seeing, it can even enhance the visual experience [1,2], in addition proper lighting can significantly cut down energy consumptions [3], but on the same time it has detrimental effects on exhibits, especially daylight. In order to safeguard the exhibits from damages, national and international standards provide specific recommendations for exhibits’ exposure, based on their photosensitivity category. These recommendations are the annual luminous exposure(LO) and the Maximum Illuminance Level (E_max_), museums’ curators have to verify that the display lighting conditions comply with the standards. Historical buildings are often converted into museums but, as their original purpose was different, the lighting conditions are often inadequate (e.g. too much uncontrolled daylight), therefore the lighting conditions’ adequacy of the space should be assessed [4]. As the name suggest the annual luminous exposure requires an annual monitoring campaign, unfortunately it often happens that exhibits have been exposed incorrectly for prolonged periods, and therefore it is very important to evaluate the need of a fast intervention. In this casuistry a prolonged measurement campaign is not acceptable. Simulations can help running a great number of analysis while reducing the length and expenses of a measurements campaign, however their previsions must be validated. This paper provides the data acquired through measurements and simulations inside the Cetacean Gallery of the Monumental Charterhouse of Calci, near Pisa (Tuscany Region, Italy). The data comprehends horizontal and vertical illuminance measurements, recorded on December the 6th, and simulations run in *Grasshopper* with the plugins *Honeybee+* and *Ladybug*. The data are related to the research article entitled “Application of climate-based daylight simulation to assess lighting conditions of space and artworks in historical buildings: the case study of Cetacean Gallery of the Monumental Charterhouse of Calci”, published on the Journal of Cultural Heritage [5].

**Specifications table**

**Table:** 

**Subject**	Building Engineering
**Specific subject area**	Daylight in buildings
**Type of data**	Tables, Images and Graphs
**How data were acquired**	On-site measurements with luxmeter (Delta OHM 2102.2), simulations run in *Grasshopper* with *Honeybee+* and *Ladybug*. Simulations rely on climate-based data provided by the Pisa Weather Station.
**Data format**	Raw, Analysed
**Parameters for data collection**	Illuminance values were collected in accordance with EN 12,464–1 recommendations [6], for defining measurement grid's dimensions, spacing and minimum number of points. *Grasshopper*’s simulations precision was ensured referring to the recommended *Radiance* parameters for accurate analyses [7].
**Description of data collection**	Illuminance values were measured with Delta Ohm 2102.2 lxmeter, on December the 6th from 10:25 to 12:25. Operators ensured not to cast shadows on the luxmeter sensor using an extension-cable between the sensor and the instrument. Horizontal and vertical illuminances were measured, for the horizontal values a plan placed 1.00 m above the floor level was used, vertical values were measured twice, first facing south and then north, with the sensor placed 1.50 m above the floor level. Annual climate-based simulations were run in *Grasshopper* using the environmental plugins *Honeybee+* and *Ladybug*. Simulations’ accuracy was validated though a confrontation with the on-site measurements.
**Data source location**	Natural History Museum of the University of Pisa, housed inside the Monumental Charterhouse of Calci, Pisa (Tuscany Region, Italy), geographic coordinates: 43°43′19″N, 10°31′22″E.
**Data accessibility**	Data are within this article; climate-based data (“epw” file format or others) can be free downloaded at https://energyplus.net/weather-location/europe_wmo_region_6/ITA//ITA_Pisa.161580_IWEC.
**Related research article**	“Application of climate-based daylight simulation to assess lighting conditions of space and artworks in historical buildings: the case study of Cetacean Gallery of the Monumental Charterhouse of Calci”, Authors: F. Leccese, G. Salvadori, G. Tambellini, Z.T. Kazanasmaz, Journal of Cultural Heritage [5].

## Value of the data

• The data in this article demonstrates that lighting software simulations can substitute prolonged measurement campaign, if the 3D model is well calibrated.
• Lighting designers can use these data as reference for comparing illuminance on-site measurements and simulations’ accuracy in similar contexts.
• The data can be used as a basis to further inquire about the daylighting adequacy inside the Natural History Museum.

## 1 Data

The data shown in this article are related to the research paper entitled “Application of climate-based daylight simulation to assess lighting conditions of space and artworks in historical buildings: the case study of Cetacean Gallery of the Monumental Charterhouse of Calci” [5]. The data validate simulations’ previsions with additional confrontations between the measured illuminance values and the software simulated ones; the data are referred to the Cetacean Gallery of the Charterhouse of Calci. The Gallery has a rectangular plan (110 × 7 m) divided in 21 bays by brick columns. Three of the four vertical surfaces are almost entirely windowed, leading to a ratio of window to floor area of 67%. With its net volume of 3426 m^3^ and floor area of 699 m^2^, the Gallery is the largest exhibition room of the Natural History Museum, housed inside the Monumental Charterhouse of Calci, near Pisa. The space corresponds with the monastery's ex-barn and hosts the most important cetacean skeleton collection in Italy, composed by 28 skeletons, 8 fossils, 47 life-sized and scale models, and 9 thematic areas.

**Fig. 1:**
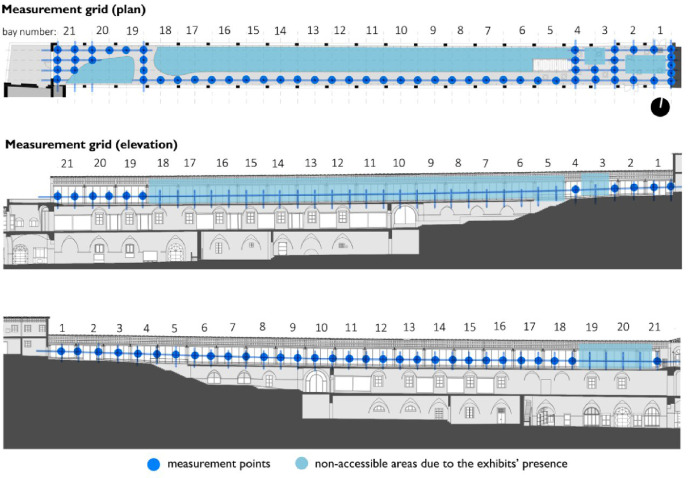
Cetacean Gallery: measurement grid (plan and elevations).

**Fig. 2:**
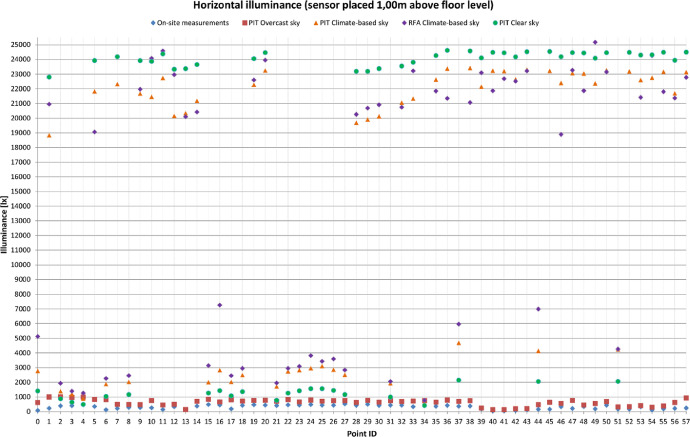
Horizontal Illuminance values on the selected points’ grid.

**Fig. 3:**
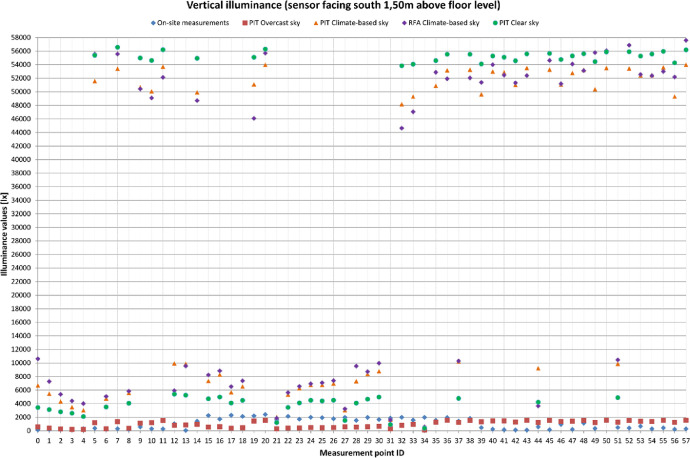
Vertical illuminance values (facing south) on the selected points’ grid.

**Fig. 4:**
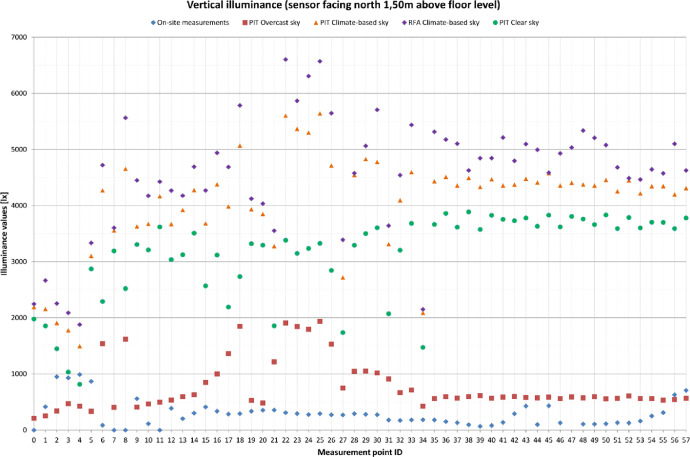
Vertical illuminance values (facing north) on the selected points’ grid.

Table 1 provides four recommended Radiance parameter settings. The settings vary depending on the level of accuracy required in the simulations. The last row of Table 1 provides a comparison with the parameter settings used in the research. Fig. 1 is a visual representation of the grids used during the measurement campaign. Floor plan and sections are displayed. Measurements points are highlighted in blue, non-accessible area (due to the exhibits’ presence) are highlighted in cyan. Table 2, Table 3 and Table 4 provide the comparison between the illuminance values that were measured on-site and those that were obtained through the simulations. Simulations results comprehends four columns of values, depending on how the results were obtained: PIT values were obtained through point-in-time analysis using Climate-Based sky (CB), CIE Clear sky and CIE Overcast sky respectively. Finally results read from annual analysis with Climate-based sky (RFA) are displayed. Fig. 2, Fig. 3, Fig. 4 are visual representations of the previous tables. The measurements points are placed on the x-axis, the illuminance levels are placed on the y-axis. Results are differentiated with symbols.

**Table 1:** Recommended *Radiance* parameters [7], depending on the required analysis accuracy.

Required analysis accuracy	ab	aa	ar	ad
Minimum	0	0.5	8	0
Fast	0	0.2	32	32
Accurate	2	0.15	128	512
Maximum	8	0.0	0	4096
**Value used in simulations**	2	0.1	300	1000

**Table 2:** Horizontal illuminance values (lx); sensor placed 1.00 m above floor level.

Grid point ID	Hour	On-site measurements	Simulations results			
			PIT Climate-based sky	RFA Climate-based sky	PIT CIE Clear sky	PIT CIE Overcast sky
**0**	12:25	82	2770	5119	1405	621
**1**	12:24	226	18,833	20,953	22,800	998
**2**	12:23	393	1384	1931	877	1001
**3**	12:23	408	1201	1387	642	966
**4**	12:22	463	886	1260	493	994
**5**	12:22	352	21,823	19,059	23,927	828
**6**	12:25	126	1876	2264	1038	812
**7**	12:24	217	22,319	25,821	24,183	495
**8**	12:23	280	2028	2456	1161	489
**9**	12:22	268	21,680	21,972	23,919	474
**10**	12:25	260	21,461	24,078	23,869	756
**11**	12:24	144	22,741	24,587	24,378	455
**12**	12:22	332	20,156	22,964	23,332	502
**13**	12:25	133	20,343	20,096	23,370	144
**14**	12:21	381	21,185	20,417	23,652	710
**15**	12:21	488	2009	3134	1261	851
**16**	12:21	450	2826	7260	1425	666
**17**	12:20	185	2026	2452	1078	810
**18**	12:20	426	2493	2948	1360	725
**19**	12:20	471	22,277	22,603	24,050	762
**20**	12:20	443	23,253	23,951	24,471	777
**21**	12:19	413	1713	1955	771	708
**22**	12:19	462	2747	2946	1258	821
**23**	12:19	446	2825	3083	1416	665
**24**	12:18	482	2953	3815	1566	790
**25**	12:18	437	3098	3427	1564	715
**26**	12:18	432	2850	3590	1443	746
**27**	12:18	527	2515	2828	1161	748
**28**	12:17	421	19,686	20,255	23,193	630
**29**	12:17	503	19,916	20,681	23,194	766
**30**	12:16	422	20,126	20,910	23,376	651
**31**	12:16	438	1934	2057	993	745
**32**	12:16	432	21,056	20,740	23,553	695
**33**	12:16	340	21,333	23,222	23,804	724
**34**	12:15	427	630	770	419	754
**35**	12:15	363	22,628	21,843	24,269	649
**36**	12:14	423	23,376	21,347	24,625	787
**37**	12:14	366	4689	5965	2145	696
**38**	12:14	380	23,418	21,065	24,579	746
**39**	12:09	186	22,136	23,095	24,106	246
**40**	12:09	50	23,241	21,866	24,486	134
**41**	12:08	52	23,210	22,684	24,451	131
**42**	12:08	77	22,644	22,518	24,172	198
**43**	12:08	128	23,314	23,219	24,529	206
**44**	12:12	159	4143	6990	2052	478
**45**	12:09	166	23,224	24,535	24,542	637
**46**	12:12	332	22,402	18,890	24,186	559
**47**	12:10	211	23,068	23,271	24,467	760
**48**	12:13	349	23,044	21,872	24,441	456
**49**	12:13	186	22,367	25,171	24,086	556
**50**	12:13	438	23,266	23,155	24,462	688
**51**	12:11	173	4235	4262	2061	323
**52**	12:11	169	23,187	24,499	24,487	339
**53**	12:11	327	22,603	21,418	24,303	413
**54**	12:10	110	22,755	24,259	24,318	300
**55**	12:11	184	23,161	21,805	24,493	387
**56**	12:10	217	21,691	21,370	23,940	624
**57**	12:11	246	23,132	22,779	24,503	929
**Average illuminance [lx]**		310	14,653	15,084	15,416	625
**MD [%]**		/	+46.2	+47.6	+48.7	+1.01

**Table 3:** Vertical illuminance values (lx); sensor facing south 1.50 m above floor level.

Grid point ID	Hour	On-site measurements	Simulations results			
			PIT Climate-based sky	RFA Climate-based sky	PIT CIE Clear sky	PIT CIE Overcast sky
**0**	10:23	146	6690	10,613	3432	583
**1**	10:24	207	5467	7277	3122	403
**2**	10:25	188	4324	5374	2796	273
**3**	10:26	213	3494	4402	2585	200
**4**	10:26	304	3021	4003	2102	191
**5**	10:26	378	51,589	55,561	55,387	1198
**6**	10:23	207	4774	5062	3512	303
**7**	10:24	306	53,412	55,578	56,565	1333
**8**	10:25	186	5592	5837	4063	370
**9**	10:27	572	50,682	50,418	54,980	1126
**10**	10:23	293	50,070	49,093	54,614	1200
**11**	10:24	270	53,702	52,120	56,207	1501
**12**	10:28	1041	9944	5921	5393	810
**13**	10:23	49	9889	9553	5253	861
**14**	10:28	1412	49,932	48,696	54,935	950
**15**	10:47	2253	7370	8233	4729	529
**16**	10:47	1718	8295	8840	4978	597
**17**	10:48	2289	5690	6515	4090	381
**18**	10:48	2117	6543	7368	4489	450
**19**	10:48	2195	51,112	46,082	55,087	1440
**20**	10:49	2406	53,992	55,703	56,299	1535
**21**	10:51	1606	2052	1868	1211	308
**22**	10:51	2137	5352	5634	3442	403
**23**	10:52	1714	6304	6547	4111	437
**24**	10:52	1985	6751	6941	4495	478
**25**	10:52	1928	6766	7084	4409	452
**26**	10:53	1772	6977	7390	4506	475
**27**	10:53	1983	3031	3229	1522	595
**28**	10:53	1530	7321	9544	4086	527
**29**	10:53	1961	8374	8718	4666	602
**30**	10:54	1655	8786	9968	4974	653
**31**	11:34	1858	1629	1591	921	306
**32**	11:35	1989	48,169	44,638	53,838	794
**33**	11:35	1573	49,285	47,050	54,070	948
**34**	11:35	1971	551	566	375	103
**35**	11:36	1525	50,881	52,875	54,592	1260
**36**	11:36	1940	53,174	51,928	55,532	1559
**37**	11:36	1415	10,228	10,298	4780	1228
**38**	11:36	1811	53,273	52,044	55,536	1523
**39**	11:39	467	49,627	51,386	54,101	1321
**40**	11:40	235	52,998	53,999	55,284	1462
**41**	11:40	161	52,866	52,464	55,095	1462
**42**	11:40	119	51,012	51,325	54,573	1283
**43**	11:40	110	53,509	52,391	55,590	1552
**44**	11:39	581	9224	3664	4219	1215
**45**	11:41	162	53,285	54,623	55,662	1543
**46**	11:39	942	51,082	51,182	54,772	1359
**47**	11:41	203	52,774	54,094	55,294	1417
**48**	11:38	1127	53,248	53,128	55,612	1527
**49**	11:38	337	50,368	55,788	54,456	1219
**50**	11:38	1527	53,512	56,063	55,870	1562
**51**	11:42	484	9885	10,459	4884	1261
**52**	11:43	411	53,441	56,871	55,913	1513
**53**	11:43	673	52,404	52,554	55,268	1406
**54**	11:42	286	52,467	52,383	55,566	1358
**55**	11:43	427	53,591	52,994	55,967	1550
**56**	11:42	225	49,300	52,176	54,270	1240
**57**	11:44	284	53,995	57,583	56,186	1567
**Average illuminance [lx]**		1032	29,881	30,264	30,349	960
**MD [%]**		/	+28.0	+28.3	+28.4	+0.07

**Table 4:** Vertical illuminance values (lx); sensor facing north 1.50 m above floor level.

Grid point ID	Hour	On-site measurements	Simulations results			
			PIT Climate-based sky	RFA Climate-based sky	PIT CIE Clear sky	PIT CIE Overcast sky
**0**	/	/	2193	2247	1980	214
**1**	10:31	416	2160	2667	1857	255
**2**	10:31	950	1912	2256	1451	342
**3**	10:31	931	1778	2090	1034	473
**4**	10:30	990	1497	1880	817	425
**5**	10:30	870	3102	3336	2873	336
**6**	10:32	87	4272	4722	2293	1540
**7**	/	/	3558	3604	3192	407
**8**	/	/	4658	5565	2523	1621
**9**	10:29	561	3632	4451	3308	411
**10**	10:32	114	3676	4175	3211	467
**11**	/	/	4167	4426	3620	500
**12**	10:29	389	3673	4268	3038	537
**13**	10:33	204	3923	4177	3126	597
**14**	10:29	303	4278	4692	3510	631
**15**	10:58	412	3686	4271	2571	850
**16**	10:58	337	4380	4940	3120	999
**17**	10:58	286	3986	4688	2193	1366
**18**	10:57	294	5070	5785	2736	1847
**19**	10:57	337	3937	4123	3323	532
**20**	10:57	355	3852	4036	3295	487
**21**	10:57	358	3280	3553	1858	1217
**22**	10:56	311	5605	6603	3384	1909
**23**	10:56	295	5370	5868	3150	1845
**24**	10:56	278	5302	6307	3237	1796
**25**	10:56	293	5643	6572	3328	1939
**26**	10:55	274	4714	5647	2847	1533
**27**	10:55	271	2723	3391	1738	751
**28**	10:54	294	4547	4578	3294	1048
**29**	10:54	283	4830	5064	3502	1051
**30**	10:54	276	4779	5708	3605	1019
**31**	11:51	180	3315	3643	2073	912
**32**	11:51	173	4097	4543	3207	670
**33**	11:50	182	4599	5438	3684	717
**34**	11:50	184	2092	2154	1474	425
**35**	11:50	182	4435	5315	3666	565
**36**	11:49	152	4513	5178	3862	596
**37**	11:49	132	4359	5105	3617	572
**38**	11:49	97	4494	4628	3889	598
**39**	11:47	69	4333	4846	3576	616
**40**	11:47	83	4470	4847	3829	570
**41**	11:47	138	4359	5213	3755	588
**42**	11:47	292	4376	4799	3732	601
**43**	11:46	428	4479	5098	3780	583
**44**	11:48	99	4413	4996	3633	575
**45**	11:46	434	4581	4588	3832	589
**46**	11:48	129	4359	4931	3622	563
**47**	11:46	596	4409	5036	3808	592
**48**	11:48	109	4377	5339	3761	575
**49**	11:48	108	4357	5208	3663	596
**50**	11:48	114	4457	5079	3834	560
**51**	11:45	132	4255	4681	3592	568
**52**	11:45	128	4451	4489	3788	609
**53**	11:45	161	4218	4465	3602	565
**54**	11:45	252	4347	4646	3704	563
**55**	11:44	313	4349	4575	3702	534
**56**	11:46	630	4199	5102	3591	546
**57**	11:44	708	4312	4627	3781	569
**Average illuminance [lx]**		293	4055	4556	3122	775
**MD [%]**		/	+12.9	+14.6	+9.67	+1.65

## 2 Experimental design, materials, and methods

Illuminance levels were measured inside the Cetacean Gallery of the Charterhouse of Calci using Delta Ohm 2102.2 lxmeter on December the 6th from 10:25 to 12:25. The measurement grid was defined according to EN 12,464–1 recommendations [6] based on the room's geometry: as the Gallery measures 110 × 7 m the standard sets the minimum number of points to 20 and their spacing to 5.0 m. The grid used for the on-site measurements is composed by 58 points, spaced 3.00 × 1.75 m (Fig. 1). Horizontal Illuminance was measured on a horizonal plane placed 1.00 m above floor level, vertical illuminance was measured 1.50 m above floor level both in north and south direction. The measured values (Table 2, Table 3, Table 4) were then confronted with software simulations (Fig. 2, Fig. 3, Fig. 4) in order to verify their accuracy. Simulations’ grid was denser, with a total of 287 measurements points spaced 1.50 × 1.75 m. However, to maximize the confrontation's significance, some points were excluded and just the exact same ones of the on-site grid were used for the confrontation. Simulations were run in *Grasshopper* using *Honeybee+* and *Ladybug, Radiance*-based environmental plugins (Table 1). The 3D model of the Gallery is based on an architectural survey conducted by the University of Pisa, the geometries were modelled in *Rhinoceros* and then imported in *Grasshopper*. Two kinds of simulation were run: point-in-time (PIT) and annual. PIT simulations are implemented in every lighting software, Annual ones are not. Annual analyses are more accurate, on account of using climate-based data for the sky model creation: the climate-based data provides the TMY (typical meteorological year) for the examined site. The TMY is composed by the succession of the most recurrent weather conditions observed during the recording period [8]. For the data used in simulations the recording period is 1982–1997. The mean deviation (MD) is calculated as (Table 2, Table 3, Table 4):

(1) $$MD=\frac{\sum_{i=1}^{n}E_{S,\;i}-\sum_{i=1}^{n}E_{M,\;i}}{\sum_{i=1}^{n}E_{M,\;i}}\%$$

where: i (1, 2, …, n) are the grid points, E_S,i_ the illuminance values obtained through the simulations, E_M,i_ the illuminance values obtained through the on-site measurements.

## 3 Funding

This research was partially funded by the 10.13039/501100007514University of Pisa as part of the biennial project: 〈*Technical committee for the predisposition of cognitive studies aimed to the restoration, the conservation and the enhancement of the Charterhouse of Calci and its Museums*〉 (2017–2019), University of Pisa board resolution N°7/2017, concerning thermal, acoustic and lighting analysis. The project involves the University of Pisa (Technical Office for the Management and the Maintenance Activities on the building heritage, and School of Engineering), the Italian Ministry of Cultural Heritage and Activities, the Italian Heritage Protection Department.

## List of abbreviations

 

ab: ambient bounces, it sets the number of diffuse bounces computed in the indirect illuminance calculation
aa: ambient accuracy, it influences the error from indirect illuminance interpolation
ar: ambient resolution, it sets the density of the ambient for the interpolation
ad: ambient divisions, it influences Monte Carlo's error during indirect illuminance calculation
E_S,i_: illuminance values obtained through the simulations
E_M,i_: illuminance values obtained through the on-site measurements
MD: mean deviation, deviation between simulated and measure illuminance values
PIT: Point In Time, illuminance values obtained through point in time analyses simulations
RFA: Read From Annual, Illuminance values obtained through annual, climate-based simulations

## Declaration of Competing Interest

The authors declare that they have no known competing financial interests or personal relationships which could have influenced the work reported in this article.
